# Disparities in Breast-Conserving Therapy for Non-Hispanic American Indian/Alaska Native Women Compared with Non-Hispanic White Women

**DOI:** 10.1245/s10434-021-10730-7

**Published:** 2021-09-06

**Authors:** Jennifer Erdrich, Felina Cordova-Marks, Angela R. Monetathchi, Manxia Wu, Arica White, Stephanie Melkonian

**Affiliations:** 1 Division of Surgical Oncology, Department of Surgery, College of Medicine, University of Arizona, Tucson, USA; 2 Department of Health Promotion Sciences, College of Public Health, University of Arizona, Tucson, USA; 3 University of Arizona, Tucson, USA; 4 Division of Cancer Prevention and Control, Centers for Disease Control and Prevention, Atlanta, USA; 5 Division of Cancer Prevention and Control, Centers for Disease Control and Prevention, Albuquerque, USA

## Abstract

**Background:**

Little is known about the surgical patterns of American Indian/Alaska Native (AI/AN) breast cancer patients. The purpose of this study is to determine whether there are disparities in breast cancer surgery and radiation therapy between non-Hispanic AI/AN (NH-AI/AN) women and non-Hispanic White (NHW) women.

**Methods:**

Data from the National Program of Cancer Registries of the Centers for Disease Control and Surveillance, Epidemiology, and End Results were used for this cross-sectional study. Female patients with invasive breast cancer diagnosed 2010–2015 were stratified by race/ethnicity, surgical procedure, radiation, and region. Percentage distributions of mastectomy and lumpectomy were compared overall and by region and stage.

**Results:**

From 2010 to 2015 there were 3292 NH-AI/AN women and 165,225 NHW women diagnosed with breast cancer. For early stage (AJCC stage 1 and 2), NH-AI/AN women had overall significantly higher percentage of mastectomy (41% vs 34.4%, *p *< 0.001) and significantly lower percentage of lumpectomy (59% vs 65.6%) compared with NHW women, without significant differences in post-lumpectomy radiation (71% vs 70%). There were regional variations, notably in the Northern Plains, where the percentage of mastectomy for early-stage disease was 48.9% for NH-AI/AN women versus 35.9% for NHW women, and in Alaska with 47% for NH-AI/AN women versus 33.3% for NHW women (*p *< 0.001). There were no overall significant differences in type of surgery or radiation for late-stage disease between groups.

**Conclusion:**

This is the first study to show disparities in surgical management of NH-AI/AN women with breast cancer. For early-stage disease, NH-AI/AN women undergo a higher percentage of mastectomy. Future clinical directions could focus on the factors that drive awareness, decision-making, and access to breast conservation.

**Supplementary Information:**

The online version contains supplementary material available at 10.1245/s10434-021-10730-7.

AI/AN women currently have the worst breast cancer survival outcomes amongst any racial group in the USA.^1–5^ Randomized trials with long-term follow-up have demonstrated that although recurrence is higher after lumpectomy alone, the survival is equivalent for patients treated with mastectomy or breast-conserving therapy (BCT), which consists of lumpectomy followed by radiation.^6–10^ This has established a surgical choice for women, but disparities persist in surgical procedures performed for different groups, even after controlling for stage of disease.^11^ BCT has been consistently found to have decreased complications and pain and better recovery and quality of life.^12–16^ There is evidence that women from racial minority groups have lower rates of BCT, breast reconstruction, and contralateral prophylactic mastectomy.^11,17–20^ While data exist for other groups, surgical patterns for AI/AN women have not been examined.

The purpose of this study is to provide a descriptive overview of surgical disparities between AI/AN and White women by US geographic region. Therefore, we evaluated the difference in distribution of surgical procedure (mastectomy and lumpectomy), as well as BCT (lumpectomy plus radiation) between the two populations by region and patient characteristics utilizing cancer registry data that has been linked with the Indian Health Service (IHS) patient registration database for the purposes of reducing racial misclassification in AI/AN populations.^21,22^

## Methods

We utilized data from population-based central cancer registries participating in the National Program of Cancer Registries of the Centers for Disease Control and Prevention (CDC) and Surveillance, Epidemiology, and End Results (SEER) program of the National Cancer Institute (NCI).^23,24^ Because the study did not involve human participants, institutional review board approval was not necessary.

Women diagnosed with primary invasive breast cancer defined by World Health Organization (WHO) International Classification of Diseases for Oncology, 3rd edition [ICD-O-3: C50.0-C50.9 excluding lymphomas, leukemia, and Kaposi sarcomas (9050-9055, 9140, 9590-9992)] from 2010 to 2015 were included.^25^ AJCC staging information was available only during these diagnosis years.^26^ Cases reported through autopsy or death certificate only, those with unknown or missing surgical status, or where radiation treatment information was not available in the database (Connecticut, Hawaii, Iowa, and New Mexico) were excluded (total AI/AN *N* = 619). Cancer cases in this study period have met the standard for high-quality data according to the United States Cancer Statistics.^27^

**Fig. 1.**
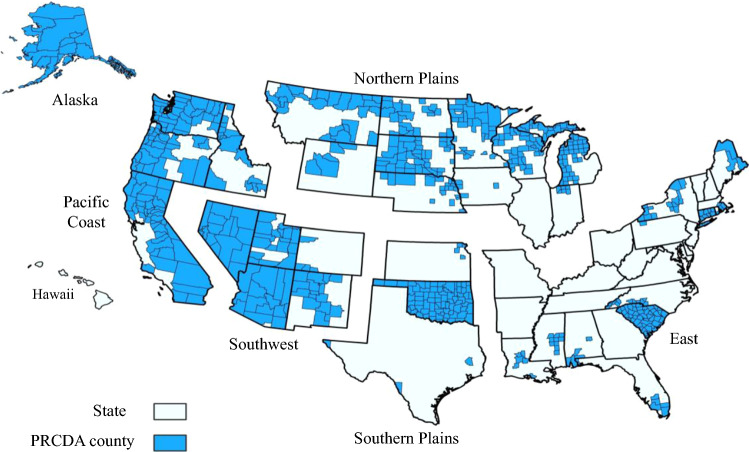
Geographic regions and purchased/referred care delivery area^a^ counties by region. ^a^Counties that contain federally recognized tribal lands or are adjacent to tribal lands. Race classification for the AI/AN population is more accurate in these counties. Percent regional coverage of AI/AN in PRCDA counties to AI/AN in all counties: northern plains 54.2%; Alaska 100%; southern plains 56.5%; southwest 83.8%; Pacific coast 60.2%; East 16.4; Total US 53.0%

Efforts to reduce racial misclassification of the AI/AN population in cancer databases have been described elsewhere.^21^ Briefly, all cases from each registry were linked with the IHS patient registration database to identify AI/AN cases with race misclassified as non-AI/AN. These linkages were conducted using LinkPlus, a probabilistic software program developed by the CDC that utilizes key patient identifiers (social security number, first name, last name, date of birth, etc.).^28^ In an effort to further improve race classification, this study focuses on IHS Purchased/Referred Care Delivery Area (PRCDA) counties.^21,22^ These counties contain or are located adjacent to federally recognized lands where AI/AN women are more likely to access IHS services. Linkages in these areas provide more accurate correction for AI/AN racial misclassification for a portion of the AI/AN population (Fig. 1). During previous analyses, it was discovered that updated bridged intercensal population estimates substantially overestimated AI/AN populations of Hispanic origin.^29^ To avoid underestimating incidence in AI/AN populations, we limited analyses to non-Hispanic AI/AN populations. The non-Hispanic White population was chosen as the referent. For conciseness, hereafter, non-Hispanic AI/AN is abbreviated as NH-AI/AN, and non-Hispanic White as NHW in reference to the study data.

Stage at diagnosis, using AJCC 7th edition was categorized into five groups: stage I, II, III, and IV and unknown stage.^26^ For this analysis, stage was further categorized into early (AJCC stage I and II) versus late (AJCC stage III, IV) because these groupings are clinically relevant for management and prognosis. Tumor subtype was classified based on estrogen receptor (ER) and progesterone receptor (PR) status. ER and PR status were combined into one of three categories (combination of collaborative stage (CS) site-specific factor 1 and CS site-specific factor 2): hormone positive (cases with ER+ or PR+ or borderline ER or PR), hormone negative (ER—and PR—cases), and unknown hormone receptor status. Unknown status included ER− cases where PR test results were unknown/missing, PR− cases where ER test results were unknown/missing, and cases where both ER and PR test results were unknown or missing. Human epidermal growth factor/neu receptor (HER2) data were not sufficient for evaluation. Tumor size was categorized as < 2 cm, 2 to < 5 cm, and > 5 cm according to the “CS Tumor Size” variable. Lymph node status was categorized as positive, negative, or unknown based on the combination of “CS Lymph Codes” or “Regional Lymph Nodes Examined.” Surgical treatment was categorized based on SEER surgery codes, RX Summ Surgery Primary Sites.

### Statistical Analysis

Average annual age-adjusted incidence rates were calculated using the direct method.^30^ Rates are expressed per 100,000 and adjusted by 19 age groups to the 2000 US standard population (Census P25-1130).^31^ Rate ratios (RR) with 95% confidence intervals (CI) were calculated for comparison of incidence rates between NH-AI/AN and NHW populations, overall and regional, according to methods described by Tiwari et al. using SEER*Stat software 8.3.2.^32,33^ Denominators for rate calculations were derived from population estimates from the US Bureau of the Census. Data were suppressed when fewer than six cases were reported. SEER*Stat was used to calculate frequencies and percentage distributions of patient characteristics including age (< 50 years, 50–69 years, 70+ years), stage, operation, radiation, lymph node status, ER/PR status, and tumor size, between NH-AI/AN and NHW populations by regions. Tests for statistical significance (chi-squared) were calculated using SAS version 9.4. Long-term trends in mastectomy by race for the years 2005–2015 were estimated by joinpoint regression with software developed by the NCI (Joinpoint Regression Program, version 4.7.0).^34^

## Results

From 2010 to 2015 there were a total of 3292 NH-AI/AN women and 165,225 NHW women diagnosed with breast cancer (Table 1). The majority who underwent lumpectomy had early-stage disease (94% NH-AI/AN women, 95.7% NHW women). There were near-equal distributions of postlumpectomy radiation (68.9% NH-AI/AN women, 68.3% NHW women). Supplementary Table 1 presents more detailed characteristics by surgery status.

**Table 1:** Individual characteristics by surgery^A^ status, overall US, female breast^B^ cancer, PRCDA counties only, Non-Hispanic American Indian and Alaska native^C^ versus Non-Hispanic white 2010–2015

	Surgery status							
	Lumpectomy				Mastectomy			
	NH AI/AN		NHW		NH AI/AN		NHW	
	N	%	N	%	N	%	N	%
Overall	1757	–	100,328	–	1535	–	64,897	–
*Age (years)*								
< 50	323	18.4	12,379	12.3	467	30.4	16,778	25.9
50-69	1055	60.0	55,816	55.6	798	52.0	32,235	49.7
70+	379	21.6	32,133	32.0	270	17.6	15,884	24.5
*Stage (early vs late)*^*D*^								
Early stage	1652	94.0	96,016	95.7	1149	74.9	50,443	77.7
Late stage	105	6.0	4312	4.3	386	25.1	14,454	22.3
*ER/PR status*^*E*^								
Positive	1466	83.4	86,574	86.3	1191	77.6	51,886	79.9
Negative	247	14.1	11,217	11.2	310	20.2	11,113	17.1
Unknown/untested/NA	44	2.5	2537	2.5	34	2.2	1,898	2.9
*Tumor size (cm)*^*F*^								
< 2	1136	64.7	72,915	72.7	583	38.0	27,972	43.1
2 to 5	571	32.5	25,302	25.2	700	45.6	27,002	41.6
5 +	46	2.6	1926	1.9	235	15.3	9208	14.2
Unknown	–^I^	–^I^	185	0.2	17	1.1	715	1.1
*Lymph node status*^*G*^								
Positive	365	20.8	16,688	17.6	704	45.90	24,749	40.7
Negative	1162	66.1	69,188	68.5	682	44.40	32,723	49.0
Unknown	230	13.1	14,452	13.8	149	9.70	7425	10.2
*Radiation status*^*H*^								
Yes	1210	68.9	68530	68.3	373	24.3	14728	22.7
No	510	29.0	30050	30.0	1129	73.6	49095	75.7
Unknown	37	2.1	1748	1.7	33	2.1	1074	1.7

*Source*: Cancer registries in the centers for disease control and prevention's national program of cancer registries (NPCR) and/or the national cancer institute's surveillance, epidemiology and end results program (SEER)

Years of data and registries used: 2010-2015 (48 states): AK*, AL*, AZ*, CA*, CO*, CT*, DE, DC, FL*, GA, HI, IA*, ID*, IL, IN*, KS*, KY, LA*, MA*, MD, ME*, MI*, MN*, MO, MT*, ND* NE*, NH, NJ, NM*, NV*, NY*, NC*, OH, OK*, OR*, PA*, RI*, SC*, TX*, TN, UT*, VT, VA, WA*, WI*, WV, WY*; 2000-2015: AR, SD*; 2003-2015: MS*. *States with at least one county designated as PRCDA.

Percent regional coverage of AI/AN in PRCDA counties to AI/AN in all counties: Northern Plains=54.2%; Alaska=100%; Southern Plains=56.5%; Southwest=83.8%; Pacific Coast=60.2%; East=16.4%; Total US=53.0%.

^A^Surgical treatment according to SEER surgery codes, breast, *RX summ-surg prim site* 01–24 (lumpectomy), 30–80 (mastectomy), code 00 and > 80 excluded from analysis, total AI/AN cases=3292, total white cases = 165,225

^B^Breast cancers in women only, only cancer or first primary only.

^C^AI/AN race is reported by NPCR and SEER registries or through linkage with the HIS patient registration database. Includes only AI/AN of non-Hispanic origin.

^D^AJCC 7 staging, early stage: I, II. late stage; III, IV; excluding pagets disease and unknown stage

^E^Combined estrogen/progesterone receptor status; variables CS site specific factor 1, CS site specific factor 2. If either listed as "positive" combined variable equals positive

^F^Tumor size derived from CS tumor size

^G^Combined *regional nodes positive* and *CS lymph node;* if either positive then coded as positive

^H^Radiation status is combined variable: RX SUMM—radiation and RAD—regional RX modality

^I^Data suppressed if counts were less than 6

*PRCDA* indicates purchased/referred care delivery areas; *NH AI/AN* non-Hispanic American Indians/Alaska natives; *NHW* non-Hispanic white

Breast cancer incidence rates by region and stage are described in Table 2. NH-AI/AN women had a statistically significant lower incidence of breast cancer compared with NHW women, all stages and all regions combined (RR 0.90, 95% CI 0.87–0.93). Incidence rates varied by region, with NH-AI/AN women from Alaska (RR 1.33, CI 1.18–1.50) and Southern Plains (RR 1.28, CI 1.20–1.38) having significantly higher incidence, while NH-AI/AN women from the Pacific Coast (RR 0.89, CI 0.83–0.96), East (RR 0.63, CI 0.54–0.67), and Southwest (RR 0.57, CI 0.51–0.62) had significantly lower incidence. For early-stage disease, incidence was significantly lower for NH-AI/AN women compared with NHW women, all regions combined (RR 0.86, CI 0.83–0.90). Incidence of late-stage disease was higher for NH-AI/AN women, all regions combined (RR 1.08, CI 1.01–1.17).

**Table 2:** Incidence rates for female breast cancer^A^ 2010–2015, Non-Hispanic American Indians/Alaska natives^B^ compared to Non-Hispanic whites for the United States, All ages overall and by AJCC stage^C^, PRCDA counties US, 2010–2015

	AI/AN rate^d^	White rate	Rate ratio^E^ (confidence interval)	*p*-value
*Total*				
Overall	89.0	99.2	0.90 (0.87–0.93)	<0.001
Northern plains	99.7	95.0	1.04 (0.97–1.14)	0.24
Alaska	128.9	96.7	1.33 (1.18–1.50)	<0.001
Southern plains	112.2	87.7	1.28 (1.20–1.38)	<0.001
Pacific coast	90.9	102.1	0.89 (0.83–0.96)	0.001
East	66.0	105.6	0.63 (0.54–0.67)	<0.001
Southwest	49.9	88.7	0.57 (0.51–0.62)	<0.001
*Early stage*				
Overall	71.9	83.4	0.86 (0.83–0.90)	<0.001
Northern plains	79.4	79.9	0.99 (0.91–1.09)	0.92
Alaska	103.6	78.4	1.32 (1.15–1.51)	0.001
Southern plains	91.0	70.5	1.24 (1.06–1.44)	<0.001
Pacific coast	75.7	86.1	0.88 (0.81–0.95)	0.001
East	55.3	89.5	0.62 (0.53–0.72)	<0.001
Southwest	38.1	74.1	0.51 (0.46–0.57)	<0.001
*Late stage*				
Overall	16.9	15.6	1.08 (1.01–1.17)	0.05
Northern plains	20.3	15.0	1.35 (1.11–1.63)	0.004
Alaska	25.1	18.2	1.38 (1.04–1.81)	0.02
Southern plains	21.0	17.0	1.24 (1.06–1.44)	0.001
Pacific coast	14.8	15.8	0.94 (0.78–1.11)	0.53
East	10.8	15.9	0.68 (0.47–0.94)	0.02
Southwest	11.8	14.5	0.81 (0.66–0.99)	0.04

*Source*: Cancer registries in the centers for disease control and prevention's national program of cancer registries (NPCR) and/or the national cancer institute's surveillance, epidemiology and end results program (SEER)

Years of data and registries used: 1999–2015 (48 states): AK*, AL*, AZ*, CA*, CO*, CT*, DE, DC, FL*, GA, HI, IA*, ID*, IL, IN*, KS*, KY, LA*, MA*, MD, ME*, MI*, MN*, MO, MT*, ND* NE*, NH, NJ, NM*, NV*, NY*, NC*, OH, OK*, OR*, PA*, RI*, SC*, TX*, TN, UT*, VT, VA, WA*, WI*, WV, WY*; 2000-2015: AR, SD*; 2003-2015: MS*. *States with at least one county designated as PRCDA.

Percent regional coverage of AI/AN in PRCDA counties to AI/AN in all counties: Northern plains = 54.2%; Alaska = 100%; Southern plains = 56.5%; Southwest = 83.8%; Pacific coast = 60.2%; east=16.4%; total US = 53.0%.

*PRCDA* indicates purchased/referred care delivery areas; *NH AI/AN* American Indians/Alaska natives; *NHW* non-Hispanic white

^A^Breast cancers in women only, only cancer or first primary only.

^B^AI/AN race is reported NPCR SEER registries or through linkage with the HIS patient registration database. Includes only AI/AN of non-Hispanic origin.

^C^AJCC staging. Early stage = AJCC stage I, II; Late stage = AJCC stage III, IV; Pagets disease and unknown stage excluded

^D^Rates are per 100,000 persons and are age-adjusted the 2000 U.S. standard (19 age groups—census P25-1130).

^E^Rate ratios (RR) are AI/AN versus White and are calculated in SEER*Stat prior to rounding of rates and may not equal RR calculated rates presented in table.

Distribution of surgical treatment by stage, region, and race is presented in Table 3. For early-stage breast cancer, a significantly higher percentage of NH-AI/AN women were treated with mastectomy (41% versus 34.4%, *p* < 0.001). For late-stage breast cancer, there was no significant differences in type of surgery performed. In the Northern Plains, 48.9% of NH-AI/AN women underwent mastectomy for early-stage disease, compared with 35.9% for NHW women (*p* < 0.001) (Table 3). In Alaska, the percentages were 47% for NH-AI/AN women versus 33.3%% for NHW women (*p* < 0.001). There were no significant differences in distribution of early-stage mastectomy in the remaining regions.

**Table 3:** Surgical treatment^A^ and radiotherapy for female breast cancer by AJCC stage^B^, Non-Hispanic American Indian and Alaska native^C^ versus Non-Hispanic white, PRCDA counties only, by region 2010–2015

	Early stage					Late stage				
	NH AI/AN		NHW		*p*-value	NH AI/AN		NHW		*p*-value
	Count	%	Count	%		Count	%	Count	%	
Overall										
Surgical treatment										
Lumpectomy	1652	59.0	96,016	65.6		105	21.4	4312	23.0	
Mastectomy	1149	41.0	50,443	34.4	<0.001	386	78.6	14,454	77.0	0.41
Radiation status for lumpectomy^D^										
Lumpectomy with radiation	1148	71.0	66,076	70.0		62	60.2	2471	58.6	
Lumpectomy without Radiation	469	29.0	28,319	30.0	0.69	41	39.8	1745	41.4	0.95
Northern plains										
Surgical treatment										
Lumpectomy	250	51.1	13,408	64.1		19	19.2	621	22.7	
Mastectomy	239	48.9	7520	35.9	<0.001	80	80.8	2119	77.3	0.42
Radiation status for lumpectomy^D^										
Lumpectomy with radiation	210	84.3	10,304	77.5		15	78.9	401	65.7	
Lumpectomy without radiation	39	15.7	2984	22.5	0.04	−^E^	21.1	209	34.3	0.49
Alaska										
Surgical treatment										
Lumpectomy	151	53.0	720	66.7		11	22.0	43	27.0	
Mastectomy	134	47.0	359	33.3	<0.001	39	78.0	116	73.0	0.47
Radiation status for lumpectomy^D^										
Lumpectomy with radiation	105	71.4	411	59.9		6	54.5	16	39.0	
Lumpectomy without radiation	42	28.6	275	40.1	0.03	–^E^	–^E^	25	61.0	0.65
Southern plains										
Surgical treatment										
Lumpectomy	520	60.0	4580	59.2		33	23.9	257	19.5	
Mastectomy	347	40.0	3155	40.8	0.66	105	76.1	1061	80.5	0.22
Radiation status for lumpectomy^D^										
Lumpectomy with radiation	370	73.1	3322	75.0		16	50.0	150	59.5	
Lumpectomy without radiation	136	26.9	1165	26.0	0.91	16	50.0	102	40.5	0.59
Pacific coast										
Surgical treatment										
Lumpectomy	413	62.8	33,342	65.0		25	24.0	1419	22.1	
Mastectomy	245	37.2	17,948	35.0	0.23	79	76.0	5015	77.9	0.63
Radiation status for lumpectomy^D^										
Lumpectomy with radiation	265	65.6	22,987	70.1		14	58.3	817	58.9	
Lumpectomy without radiation	139	34.4	9813	29.9	0.15	10	41.7	569	41.1	0.99
East										
Surgical treatment										
Lumpectomy	111	64.2	32,012	68.0		–^E^	–^E^	1379	24.5	
Mastectomy	62	35.8	15,081	32.0	0.29	15	75.0	4241	75.5	0.96
Radiation status for lumpectomy^d^										
Lumpectomy with radiation	86	78.9	24,037	76.0		–^E^	–^E^	859	63.3	
Lumpectomy without radiation	23	21.1	7593	24.0	0.77	–^E^	–^E^	498	36.7	0.74
Southwest										
Surgical treatment										
Lumpectomy	207	62.9	11,954	65.2		12	15.0	596	23.8	
Mastectomy	122	37.1	6380	34.8	0.39	68	85.0	1911	76.2	0.06
Radiation status for lumpectomy^D^										
Lumpectomy with radiation	112	55.4	5015	43.6		7	58.3	228	40.0	
Lumpectomy without radiation	90	44.6	6489	56.4	0.004	−^E^	–^E^	342	60.0	0.44

Chi-squared *p*-value used to assess significant differences in distribution of surgical status by race

^A^Surgical treatment according to SEER surgery codes, breast, *RX summ-surg prim* site 01–24 (lumpectomy), 30–80 (mastectomy), code 00 and > 80 excluded from analysis

^B^AJCC 7 staging, early stage: I, II. Late Stage; III, IV; excluding pagets disease

^C^AI/AN race is reported by NPCR and SEER registries or through linkage with the HIS patient registration database. Includes only AI/AN of non-Hispanic origin. NH *AI/AN* non-hispanic AI/AN; *NHW* non-hispanic white

^D^Only included those with non-missing surgical and radiation status

^E^Data were suppressed if counts were less than 6

For those undergoing lumpectomy with complete treatment information, distribution of radiation was also calculated to assess differences in BCT (Table 3). There were no overall differences in postlumpectomy radiation between groups for early- or late-stage disease. Regional analysis showed that in the Northern Plains (84.3% versus 77.5%, *p* = 0.04), Alaska (71.4% versus 59.9%, *p* = 0.03), and Southwest (55.4% versus 43.6%, *p* = 0.004), a higher percentage of NH-AI/AN women with early-stage breast cancers received postlumpectomy radiation.

**Fig. 2.**
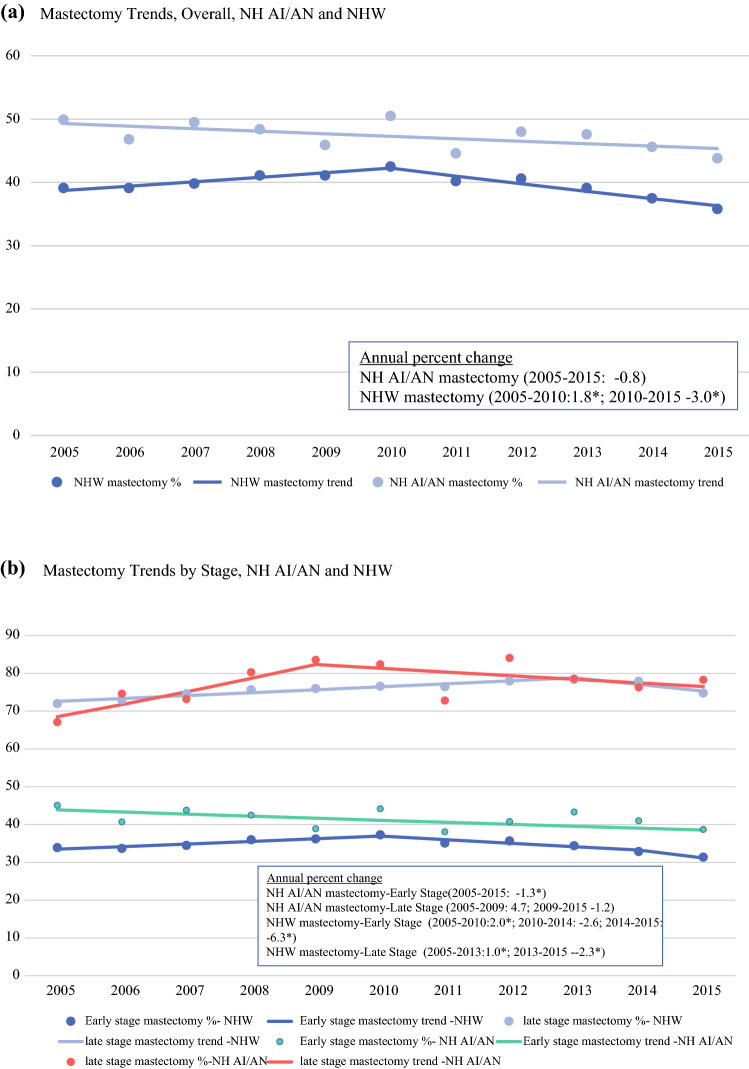
Trends in mastectomy^a^, by race and stage^b^, 2005–2015, non-Hispanic AI/AN^c^ and non-Hispanic white females, PRCDA counties: **a** mastectomy trends, overall, NH AI/AN and NHW, **b** mastectomy trends by stage, NH AI/AN and NHW. ^a^Surgical treatment according to SEER surgery codes, breast, *RX summ-surg prim* site 01–24 (lumpectomy), 30–80 (mastectomy), code 00 and > 80 excluded from analysis. ^b^AJCC 7 staging, Early stage: I, II. Late stage; III, IV; excluding Paget’s disease. ^c^AI/ANrace is reported by NPCR and SEER registries or through linkage with the HIS patient registration database. Includes only AI/AN of non-Hispanic origin

The percentage of NH-AI/AN women who underwent mastectomy was stable 2005–2015 (Fig. 2A). For NHW women, the mastectomy trend increased significantly from 2005 to 2010 (APC 1.8), then decreased significantly from 2010 to 2015 (APC—3.0). The APC for mastectomy varied by disease stage and time frame (Fig. 2B). For early-stage disease, the APC decreased for NH-AI/AN women (1.3) during the time period, but increased (2005–2010), plateaued (2010–2014), then decreased (2014–2015) for NHW women. For late-stage disease, the APC for mastectomy remained level for NH-AI/AN women but increased (2005–2013) before decreasing (2013–2015) for NHW women.

## Discussion

By using cancer registry data linked with the IHS patient registration database, we detected differences in the surgical management of breast cancer for NH-AI/AN and NHW women. NH-AI/AN women with early-stage breast cancer had significantly higher reported use of mastectomy compared with NHW women (41% versus 34.4%, *p* < 0.001). These differences were prominent for NH-AI/AN women from the Northern Plains and Alaska, where 47–49% received mastectomy compared with 33–36% of NHW women in the same region. When BCT was implemented, we found no overall differences in postlumpectomy radiation between NH-AI/AN and NHW women, but did identify three regions where NH-AI/AN women were more likely than NHW women to undergo postlumpectomy radiation, a trend seen elsewhere in lower socioeconomic groups.^35^ Disparities in breast cancer screening, stage, and morbidity/mortality have been examined previously for AI/AN women compared with the general population; ^1–5,36,37^ however, we believe this is the first study to specifically examine disparities in breast cancer surgery for AI/AN women.

### Cancer Disparities

Research has demonstrated that AI/AN patients are less likely to receive guideline-concordant cancer care related to surgery, adjuvant therapy, and surveillance.^3–5,38^ These are important factors considering that AI/AN patients have the worst cancer survival rates of any US ethnic group.^4,39^ SEER analyses have shown that AI/AN women are less likely to receive standard adjuvant chemotherapy for breast cancer,^3,5,37,38,40^ and other data have likewise shown a lower likelihood of receiving guideline-concordant preoperative biopsy, adjuvant therapy, and post-therapy surveillance, and higher likelihood of significant treatment delays.^3–5,37,40^ Endocrine therapy for breast cancer has been shown to be less optimally used in women from other minority groups, but these studies did not include AI/AN women.^41^ In a chart review of Navajo patients with breast cancer, 30% did not receive standard care, which was attributed to cultural, structural, and geographic challenges.^42,43^

### Surgical Disparities

Differences in surgery have been noted for other minority women. Hispanic and African American women have been shown to have higher mastectomy rates.^44^ Two studies of Hispanic women with early-stage disease showed lower odds of lumpectomy.^45,46^ A study of Medicare beneficiaries in Alabama found that residents with lower socioeconomic status (SES) were more likely to undergo mastectomy and postlumpectomy radiation, similar to our findings.^35^ AI/AN women have been shown to have longer lengths of stay and half the likelihood of outpatient breast surgery.^47,48^ Other studies have shown lower rates of breast reconstruction for racial minorities,^20,44,49^ but information on postmastectomy reconstruction was not available for the present study.

After a steady increase in BCT in the 1990s, a phenomenon of increased bilateral mastectomies occurred with differences related to race and SES, and a further inflection after Angelina Jolie’s highly publicized surgery in 2013.^9,50,51^ In a study on patterns of contralateral prophylactic mastectomy (CPM), White women had 50% greater likelihood of CPM compared with minority women and those privately insured had 62% greater likelihood.^52^ This trend of White and privately insured women electing mastectomy when eligible for lumpectomy might be narrowing the disparity in our study, which is noteworthy since the findings remained robust. Research has shown rurality affects reconstruction as patients from surgical deserts are significantly less likely to receive reconstruction.^49^ The lower reconstruction rates for rural women and minorities suggest that AI/AN women are similarly vulnerable.

There are documented advantages of BCT compared with mastectomy. From a systems perspective, the cost of lumpectomy is lower, particularly compared with mastectomy with reconstruction, although the radiation fees associated with BCT can variably impact the cost savings.^53,54^ From the patient’s perspective, BCT has been consistently cited to have fewer complications, less pain, faster recovery, more favorable cosmetics, and better preserved sexuality, body image, and quality of life.^6,7,12–16,55^ In consideration of the potential differences in cost and quality of life, in addition to the known equivalent survival, the higher mastectomy rate of 41% for NH-AI/AN women compared with 34% for NHW women with early-stage breast cancer is not only statistically significant but possibly clinically significant across these parameters. Addressing this disparity in the Northern Plains and Alaska where the difference is particularly prominent might have even greater clinical impact for these regions*.* AI/AN women with early-stage disease undergoing mastectomy for what might otherwise be treated with lumpectomy may be disproportionately impacted on these measures, though it must be acknowledged that selection of mastectomy might occur for clinical reasons.

### Radiation Disparities

Radiation therapy (RT) is a critical component of BCT as it lowers local recurrence compared with lumpectomy alone and provides survival outcomes comparable to mastectomy.^56^ RT is costly and delivered at specialty centers, most often urban. Conventional RT requires daily visits for up to 7 weeks, which can be prohibitive to those on rural, tribal lands.^56,57^ Prior research has shown a relationship between mastectomy and RT accessibility, with one study finding a 44% greater likelihood of mastectomy for remote patients.^57–59^ We had hypothesized that higher mastectomy for AI/AN women might be driven by lower access to RT; however, our data showed nonsignificant differences in RT between NH-AI/AN and NHW women overall, and increased utilization in three regions. We do not have detailed RT data in terms of course length, or whole-breast versus partial-breast irradiation (PBI). A study examining PBI, which can be delivered in 5 days, found that AI/AN women used PBI more than twice as often as their urban counterparts.^56^ With limited RT data available, the primary purpose of this study was to characterize differences in surgical treatment.

### Geographic Barriers

The georemote location of reservations is another factor potentially contributing to surgical disparities.^2,38^ Prior studies show longer travel time to breast imaging facilities may influence actual breast cancer treatment, wherein greater distance is associated with higher probability of mastectomy, as well as decreased postlumpectomy radiation.^11,59–64^ Distance may be contributing to NH-AI/AN women’s selection of mastectomy as it may be the better individual choice if distance to a radiation facility is prohibitive to lumpectomy. In a study of over 92 million women, AI/AN women had longer median travel times to all breast imaging modalities compared with all racial/ethnic groups.^64^ AI/AN women have been widely documented to underutilize screening services with geography a key factor.^1,36,64,65^ Compounding geography, inclement weather heightens barriers as women with greater travel distances are less likely to undergo mammogram during winter.^64,66^ Of note, the two regions in our study where NH-AI/AN women had the highest percentages of mastectomy are the Northern Plains and Alaska, which have notorious winters and rurality. There are many benefits to concentrating resources at high-volume centers; however, their urban location can adversely skew treatment for rural patients.^61^ This can be particularly exacerbated for AI/AN patients because the IHS does not have onsite oncology facilities and can only refer patients to tertiary cancer centers through Purchased Referred Care.^2,3,38^ Geographic distance becomes further problematic in that those in rural areas with less financial means, relevant for AI/AN populations, may not have transportation or time from work for extended travel to maintain treatment.^38^

### Limitations

While this study utilized the most accurate, up-to-date data for cancer incidence in NH-AI/AN populations, there are limitations. Because racial misclassification was addressed through linkage with the IHS, these corrections for misclassification applied only to persons who are members of federally recognized tribes and accessed services through the IHS. The exclusion of Hispanic AI/AN persons and data from some registries may disproportionately impact AI/AN data from certain regions. Individuals living in urban non-PRCDA areas are also not represented in this data; future analyses will be needed to address these limitations. While we evaluated several clinical characteristics, this study is descriptive in nature, and therefore we were unable to take into account potential confounding by other factors between race/ethnicity and surgical treatment. Additionally, RT data may be underreported in cancer registries.^67^ The present RT analysis is limited to those with complete data, therefore future efforts to more fully characterize RT are needed. Lastly, we did not have information regarding comorbidities or anatomic factors that might influence appropriateness of one operation over the other.

## Conclusions

This study demonstrates that NH-AI/AN women with early-stage disease are undergoing mastectomy at higher percentage than NHW women. We contribute a new and important finding that there are surgical disparities in breast cancer for NH-AI/AN women. In terms of BCT, our study also shows that, when lumpectomy is selected, it is consistently followed by radiation for both groups overall, but with key regional differences. Northern Plains, Alaska, and Southwest had statistically significant higher rates of postlumpectomy radiation for NH-AI/AN women. How these regions succeed at standard BCT for NH-AI/AN women who prefer it would be important knowledge for other service areas. Future directions in breast cancer care for AI/AN women could consider the personal and systems factors that lead to increased mastectomy and how access to BCT might be improved for those who otherwise prefer it. Partnerships between academic/tertiary centers and tribal facilities, mobile screening units, telemedicine, patient navigation, transportation assistance, utilization of accelerated or partial-breast irradiation, all with cultural and linguistic sensitivity at the forefront, are avenues to increase AI/AN access to BCT. Dismantling cancer disparities is a complex, multilevel task demanding multidisciplinary collaboration, but it is of utmost importance for AI/AN women who endure a legacy of colonization, displacement, and poverty, all of which negatively impact health including cancer detection, treatment, and survivorship.

## Disclaimer

The findings and conclusions in this report are those of the authors and do not necessarily represent the official position of the Centers for Disease Control and Prevention.

## Supplementary Information

Below is the link to the electronic supplementary material.

Supplementary file1 (DOCX 25 kb)
