# Supplementary material for: Disparities in Breast-Conserving Therapy for Non-Hispanic American Indian/Alaska Native Women Compared with Non-Hispanic White Women
Source: Ann Surg Oncol. 2021 Sep 6;29(2):1019–30. doi: 10.1245/s10434-021-10730-7 (PMC8724083; doi:10.1245/s10434-021-10730-7)
Supplement: Supplementary file 1 — Supplementary file1 (DOCX 25 kb) [file 10434_2021_10730_MOESM1_ESM.docx]

| **Supplemental Table 1: Individual Characteristics by Surgery^a^ Status, By Region, Female Breast^b^ Cancer, PRCDA Counties Only, American Indian and Alaska Native^c^ versus White 2010-2015** | | | | | | | | | | | | |
| --- | --- | --- | --- | --- | --- | --- | --- | --- | --- | --- | --- | --- |
|  | **Northern Plains** | | **Alaska** | | **Southern Plains** | | **Pacific Coast** | | **East** | | **Southwest** | |
|  | Lumpectomy | Mastectomy | Lumpectomy | Mastectomy | Lumpectomy | Mastectomy | Lumpectomy | Mastectomy | Lumpectomy | Mastectomy | Lumpectomy | Mastectomy |
|  | N (%) | N (%) | N (%) | N (%) | N (%) | N (%) | N (%) | N (%) | N (%) | N (%) | N (%) | N (%) |
| Overall |  |  |  |  |  |  |  |  |  |  |  |  |
| **Age** |  |  |  |  |  |  |  |  |  |  |  |  |
| NH AI/AN |  |  |  |  |  |  |  |  |  |  |  |  |
| <50 | 58 (21.6) | 88 (27.6) | 31 (19.1) | 53 (30.6) | 102 (18.4) | 143 (31.6) | 64 (14.6) | 102(31.5) | 23 (19.8) | 22 (28.6) | 45 (20.5) | 59 (31.1) |
| 50-69 | 170 (63.2) | 187 (58.6) | 99 (61.1) | 88 (50.9) | 319 (57.7) | 222 (49.1) | 274 (62.6) | 162(50) | 59 (50.9) | 42 (54.5) | 134 (61.2) | 97 (51.1) |
| 70+ | 41 (15.2) | 44 (13.8) | 32 (19.8) | 32 (18.5) | 132 (23.9) | 87 (19.2) | 100 (22.8) | 60(18.5) | 34 (29.3) | 13 (16.9) | 40 (18.3) | 34 (17.9) |
| NHW |  |  |  |  |  |  |  |  |  |  |  |  |
| <50 | 1711 (12.2) | 2558 (26.5) | 136 (17.9) | 127 (26.7) | 584 (12.1) | 886 (21.1) | 3976 (11.4) | 5627(24.5) | 4492 (13.5) | 5503 (28.5) | 1480 (11.8) | 2077 (25.1) |
| 50-69 | 7902 (56.3) | 4693 (48.7) | 481 (63) | 254 (53.5) | 2709 (56) | 2098 (49.8) | 19790 (56.9) | 11701(51) | 18169 (54.4) | 9375 (48.5) | 6765 (53.9) | 4114 (49.6) |
| 70+ | 4416 (31.5) | 2389 (24.8) | 146 (19.1) | 94 (19.8) | 1544(31.9) | 1232 (29.2) | 10995 (31.6) | 5635(24.5) | 10727 (32.1) | 4435 (23) | 4305 (34.3) | 2100 (25.3) |
|  |  |  |  |  |  |  |  |  |  |  |  |  |
| **Stage (Early vs Late) ^d^** | |  |  |  |  |  |  |  |  |  |  |  |
| NH AI/AN |  |  |  |  |  |  |  |  |  |  |  |  |
| Early Stage | 250 (92.9) | 239 (74.9) | 151 (93.2) | 134 (77.5) | 520(94) | 347 (76.8) | 413 (94.3) | 245(75.6) | 111 (95.7) | 62 (80.5) | 207 (94.5) | 122 (64.2) |
| Late Stage | 19 (7.1) | 80 (25.1) | 11 (6.8) | 39 (22.5) | 33(6) | 105 (23.2) | 25 (5.7) | 79(24.4) | 5 (4.3) | 15 (19.5) | 12 (5.5) | 68 (35.8) |
| NHW |  |  |  |  |  |  |  |  |  |  |  |  |
| Early Stage | 13408 (95.6) | 7520 (78) | 720 (94.4) | 359 (75.6) | 4580(94.7) | 3155 (74.8) | 33342 (95.9) | 17948(78.2) | 32012 (95.9) | 15081 (78.1) | 11954 (95.3) | 6380 (77) |
| Late Stage | 621 (4.4) | 2119 (22) | 43 (5.6) | 116 (24.4) | 257(5.3) | 1061 (25.2) | 1419 (4.1) | 5015(21.8) | 1376 (4.1) | 4232 (21.9) | 596 (4.7) | 1911 (23) |
| **ER/PR status^e^** |  |  |  |  |  |  |  |  |  |  |  |  |
| NH AI/AN |  |  |  |  |  |  |  |  |  |  |  |  |
| Positive | 215 (79.9) | 240 (75.2) | 142 (87.7) | 143 (82.7) | 449(81.2) | 348 (77) | 381 (87) | 263(81.2) | 97 (83.6) | 62 (80.5) | 182 (83.1) | 135 (71.1) |
| Negative | 47 (17.5) | 78 (24.5) | 18 (11.1) | 30 (17.3) | 85(15.4) | 91 (20.1) | 50 (11.4) | 56(17.3) | 16 (13.8) | 13 (16.9) | 31 (14.2) | 42 (22.1) |
| Unknown/ untested/NA | 7 (2.6) | -^i^ | -^i^ | -^i^ | 19(3.4) | 13 (2.9) | 7 (1.6) | -^i^ | -^i^ | -^i^ | 6 (2.7) | 13 (6.8) |
| NHW |  |  |  |  |  |  |  |  |  |  |  |  |
| Positive | 12336 (87.9) | 7835 (81.3) | 673 (88.2) | 390 (82.1) | 4025(83.2) | 3202 (75.9) | 30452 (87.6) | 18755(81.6) | 28537 (85.5) | 15285 (79.1) | 10551 (84.1) | 6419 (77.4) |
| Negative | 1607 (11.5) | 1730 (17.9) | 82 (10.7) | 80 (16.8) | 669(13.8) | 859 (20.4) | 3648 (10.5) | 3689(16.1) | 3801 (11.4) | 3330 (17.3) | 1410 (11.2) | 1425 (17.2) |
| Unknown/ untested/NA | 86 (0.6) | 74 (0.8) | 8 (1) | 5 (1.1) | 143(3) | 155 (3.7) | 661 (1.9) | 519(2.3) | 1050 (3.1) | 698 (3.6) | 589 (4.7) | 447 (5.4) |
| **Tumor Size (cm)^f^** |  |  |  |  |  |  |  |  |  |  |  |  |
| NH AI/AN |  |  |  |  |  |  |  |  |  |  |  |  |
| <2 | 157 (58.4) | 100 (31.3) | 112 (69.1) | 94 (54.3) | 362(65.5) | 161 (35.6) | 295 (67.4) | 129(39.8) | 81 (69.8) | 35 (45.5) | 129 (58.9) | 64 (33.7) |
| 2 to 5 | 102 (37.9) | 165 (51.7) | 48 (29.6) | 62 (35.8) | 169(30.6) | 222 (49.1) | 135 (30.8) | 144(44.4) | 33 (28.4) | 29 (37.7) | 84 (38.4) | 78 (41.1) |
| 5+ | 10 (3.7) | 51 (16) | -^i^ | 12 (6.9) | 21(3.8) | 65 (14.4) | 7 (1.6) | 48(14.8) | -^i^ | 12 (15.6) | -^i^ | 47 (24.7) |
| Unknown/ uncategorized | -^i^ | -^i^ | -^i^ | -^i^ | -^i^ | -^i^ | -^i^ | -^i^ | -^i^ | -^i^ | -^i^ | -^i^ |
| NHW |  |  |  |  |  |  |  |  |  |  |  |  |
| <2 | 10332 (73.6) | 4317 (44.8) | 531 (69.6) | 206 (43.4) | 3267(67.5) | 1540 (36.5) | 24895 (71.6) | 9817(42.8) | 25006 (74.9) | 8715 (45.1) | 8884 (70.8) | 3377 (40.7) |
| 2 to 5 | 3396 (24.2) | 3861 (40.1) | 212 (27.8) | 197 (41.5) | 1407(29.1) | 2025 (48) | 9132 (26.3) | 9657(42.1) | 7741 (23.2) | 7714 (39.9) | 3414 (27.2) | 3548 (42.8) |
| 5+ | 276 (2) | 1372 (14.2) | 18 (2.4) | 61 (12.8) | 151(3.1) | 601 (14.3) | 672 (1.9) | 3262(14.2) | 580 (1.7) | 2664 (13.8) | 229 (1.8) | 1248 (15.1) |
| Unknown/uncategorized | 25 (0.2) | 89 (0.9) | -^i^ | 11 (2.3) | 12(0.2) | 50 (1.2) | 62 (0.2) | 227(1) | 61 (0.2) | 220 (1.1) | 23 (0.2) | 118 (1.4) |
| **Lymph Node Status^g^** | |  |  |  |  |  |  |  |  |  |  |  |
| NH AI/AN |  |  |  |  |  |  |  |  |  |  |  |  |
| Positive | 145 (53.9) | 125 (39.2) | 113 (69.8) | 86 (49.7) | 410(74.1) | 239 (52.9) | 277 (63.2) | 145(44.8) | 72 (62.1) | 34 (44.2) | 157 (71.7) | 79 (41.6) |
| Negative | 55 (20.4) | 120 (37.6) | 37 (22.8) | 81 (46.8) | 112(20.3) | 199 (44) | 69 (15.8) | 128(39.5) | 32 (27.6) | 36 (46.8) | 36 (16.4) | 98 (51.6) |
| Unknown | 69 (25.7) | 74 (23.2) | 12 (7.4) | 6 (3.5) | 31(5.6) | 14 (3.1) | 92 (21) | 51(15.7) | 12 (10.3) | 7 (9.1) | 26 (11.9) | 13 (6.8) |
| NHW |  |  |  |  |  |  |  |  |  |  |  |  |
| Positive | 8252 (58.8) | 4030 (41.8) | 551 (72.2) | 265 (55.8) | 3647(75.4) | 2231 (52.9) | 23679 (68.1) | 11493(50.1) | 24542 (73.5) | 10491 (54.3) | 8517 (67.9) | 4213 (50.8) |
| Negative | 1967 (14) | 3068 (31.8) | 160 (21) | 184 (38.7) | 931(19.2) | 1860 (44.1) | 5635 (16.2) | 8606(37.5) | 5729 (17.2) | 7773 (40.2) | 2266 (18.1) | 3258 (39.3) |
| Unknown | 3810 (27.2) | 2541 (26.4) | 52 (6.8) | 26 (5.5) | 259(5.4) | 125 (3) | 5447 (15.7) | 2864(12.5) | 3117 (9.3) | 1049 (5.4) | 1867 (14.1) | 820 (9.9) |
| **Radiation Status^h^** |  |  |  |  |  |  |  |  |  |  |  |  |
| NH AI/AN |  |  |  |  |  |  |  |  |  |  |  |  |
| Yes | 225 (83.6) | 95 (29.8) | 111 (68.5) | 37 (21.4) | 386(69.8) | 118 (26.1) | 279 (63.7) | 63(19.4) | 90 (77.6) | 20 (26) | 119 (54.3) | 40 (21.1) |
| No | 43 (16) | 219 (68.7) | 47 (29) | 131 (75.7) | 152(27.5) | 324 (71.7) | 149 (34) | 254(78.4) | 24 (20.7) | 57 (74) | 95 (32.4) | 144 (75.8) |
| Unknown | -^i^ | -^i^ | -^i^ | -^i^ | 15(2.7) | 10 (2.2) | 10 (2.3) | 7(2.2) | -^i^ | -^i^ | -^i^ | 6 (3.2) |
| NHW |  |  |  |  |  |  |  |  |  |  |  |  |
| Yes | 10703 (76.3) | 2640 (27.4) | 426 (55.8) | 77 (16.2) | 3471(71.8) | 998 (23.7) | 23800 (68.5) | 4739(20.6) | 24891 (74.6) | 5033 (26.1) | 5239 (41.4) | 1241 (15) |
| No | 3192 (22.8) | 6904 (71.6) | 300 (39.3) | 372 (78.3) | 1267(26.2) | 3135 (74.4) | 10377 (29.9) | 15886(77.9) | 8087 (24.2) | 13979 (72.4) | 6827 (54.4) | 6819 (82.2) |
| Unknown | 134 (1) | 95 (1) | 37 (4.8) | 26 (5.5) | 99(2) | 83 (2) | 584 (1.7) | 338(1.5) | 410 (1.2) | 301 (1.6) | 484 (3.9) | 231 (2.8) |
|  |  |  |  |  |  |  |  |  |  |  |  |  |
| PRCDA indicates Purchased/Referred Care Delivery Areas; NH AI/AN: non-Hispanic American Indians/Alaska Natives; NHW: non-Hispanic white  ^a^ Surgical treatment according to SEER Surgery Codes, Breast, *RX summ-surg prim site* 01-24 (lumpectomy), 30-80 (mastectomy), Code 00 and >80 excluded from analysis, total AI/AN cases=3292, total white cases = 165,225 | | | | | | | | | | | | |
| ^b^Breast cancers in women only, only cancer or first primary only. | | | |  |  |  |  |  |  |  |  |  |
| ^c^ AI/AN race is reported by NPCR and SEER registries or through linkage with the IHS patient registration database. Includes only AI/AN of non-Hispanic origin. | | | | | | | | | | |  |  |
| ^d^ AJCC 7 staging, Early Stage: I,II. Late Stage; III, IV, V; excluding pagets disease | | | | | |  |  |  |  |  |  |  |
| ^e^ combined estrogen/progesterone receptor status; variables CS site specific factor 1, CS site specific factor 2. If either listed as "positive" combined variable equals positive | | | | | | | | | | | |  |
| ^f^ Tumor size derived from CS Tumor Size | | |  |  |  |  |  |  |  |  |  |  |
| ^g^ Combined *Regional Nodes Positive* and *CS Lymph Node;* if either positive then coded as positive | | | | | | |  |  |  |  |  |  |
| ^h^ Radiation status is combined variable: RX SUMM--RADIATION and RAD--REGIONAL RX MODALITY | | | | | | |  |  |  |  |  |  |
| ^i^Data suppressed if counts were less than 6 | | |  |  |  |  |  |  |  |  |  |  |
| Years of data and registries used: 2010-2015 (48 states): AK*, AL*, AZ*, CA*, CO*, CT*, DE, DC, FL*, GA, HI, IA*, ID*, IL, IN*, KS*, KY, LA*, MA*, MD, ME*, MI*, MN*, MO, MT*, ND* NE*, NH, NJ, NM*, NV*, NY*, NC*, OH, OK*, OR*, PA*, RI*, SC*, TX*, TN, UT*, VT, VA, WA*, WI*, WV, WY*; 2000-2015: AR, SD*; 2003-2015: MS*. *States with at least one county designated as PRCSDA. | | | | | | | | | | | | |
| Percent regional coverage of AI/AN in PRCDA counties to AI/AN in all counties: Northern Plains=54.2%; Alaska=100%; Southern Plains=56.5%; Southwest=83.8%; Pacific Coast=60.2%; East=16.4%; Total US=53.0%. | | | | | | | |  |  |  |  |  |
|  |  |  |  |  |  |  |  |  |  |  |  |  |
